# Supplementary material for: Relative Age Effect in the Sport Environment. Role of Physical Fitness and Cognitive Function in Youth Soccer Players
Source: Int J Environ Res Public Health. 2019 Aug 8;16(16):2837. doi: 10.3390/ijerph16162837 (PMC6719027; doi:10.3390/ijerph16162837)
Supplement: Supplementary file 1 [file ijerph-16-02837-s001.pdf]

Supplementary Material. Table S1. Correlations between Age, Anthropometrics, Physical Fitness and Attentional Functions per age group

		Variables	Age Group	1	2	3	4	5	6	7	8	9	10	11	12	13	14	15	16	17	18	19	20	21	22
Age & Anthropometrics		(1) Chronological Age	All		0.645***	0.566***	-0.628***	-0.578***	0.549***	0.028	-0.363***	-0.149	-0.111	0.012	-0.145	0.003	0.089	-0.084	-0.203	-0.325**	-0.212*	0.016	-0.092	-0.403***	-0.337**
			u10	-	0.101	-0.108	-0.191	-0.004	-0.002	0.107	-0.096	-0.197	0.194	-0.184	0.296	-0.154	0.264	-0.002	0.163	-0.118	0.034	-0.254	0.123	0.193	0.044
			u12		0.094	0.111	-0.261	-0.207	0.162	0.251	0.160	0.192	-0.121	-0.041	-0.189	-0.053	-0.059	0.149	-0.084	0.158	0.179	0.099	-0.031	0.114	0.368*
		(2) Height	All			0.839***	-0.383***	-0.408***	0.130	-0.034	-0.256*	-0.174	-0.021	-0.054	-0.036	-0.134	0.091	0.064	-0.049	-0.216*	-0.285**	0.027	-0.092	-0.338**	-0.250*
			u10		-	0.669***	0.150	0.032	-0.385	0.223	0.014	0.070	0.132	-0.040	0.086	-0.263	0.018	0.231	0.128	-0.009	-0.223	0.046	0.085	0.058	0.061
			u12			0.787***	-0.245	-0.162	-0.245	-0.159	-0.064	-0.311*	0.134	-0.165	0.123	-0.091	0.092	0.168	0.193	-0.008	-0.162	-0.090	-0.110	-0.169	-0.012
		(3) Weight	All				-0.217	-0.270*	0.026	-0.198	-0.126	-0.071	-0.086	-0.023	0.029	-0.078	0.036	0.050	-0.121	-0.070	-0.149	0.109	-0.028	-0.271*	-0.274*
			u10			-	0.350*	0.249	-0.386	-0.057	0.202	0.108	-0.020	-0.002	0.097	-0.272	-0.036	0.164	0.056	0.218	-0.150	0.216	0.136	0.061	-0.061
			u12				0.074	0.007	-0.489**	-0.307*	0.051	-0.085	0.063	-0.103	0.248	0.041	0.016	0.155	-0.000	0.112	0.087	-0.022	0.010	-0.062	-0.034
Physical Fitness		(4) Agility T-test	All					0.765***	-0.650***	-0.161	0.210	0.209	0.172	0.124	0.240*	-0.185	0.020	0.108	0.177	0.167	0.206	-0.001	0.133	0.289*	0.022
			u10				-	0.779***	-0.456*	-0.223	-0.047	0.142	0.116	0.127	0.238	-0.280	0.188	0.081	0.105	-0.070	0.035	0.038	0.027	0.011	-0.363*
			u12					0.514***	-0.459**	-0.333*	0.117	0.337*	-0.145	0.183	-0.042	-0.070	-0.140	-0.042	-0.255	0.177	0.391*	0.150	0.038	0.076	-0.029
		(5) 24 m Speed	All						-0.579***	-0.111	0.181	0.182	0.131	0.115	0.238*	-0.044	-0.082	0.208	0.119	0.156	0.194	-0.022	0.086	0.390***	0.055
			u10					-	-0.256	-0.180	0.070	0.239	-0.024	0.103	0.051	-0.240	0.160	0.162	-0.025	0.026	0.106	0.137	-0.046	0.090	-0.375*
			u12						-0.477**	-0.195	-0.083	0.093	0.031	0.217	0.293	0.144	-0.268	0.147	-0.128	0.003	0.171	-0.027	-0.027	0.272	-0.036
		(6) Endurance TTE	All							0.078	-0.160	-0.000	-0.221	-0.110	-0.407***	0.138	-0.097	-0.182	-0.207	-0.186	-0.218	0.131	-0.172	-0.421***	-0.233
			u10						-	-0.178	-0.071	0.032	-0.030	-0.218	-0.382	0.256	-0.108	-0.013	-0.090	-0.113	-0.057	0.029	0.023	-0.225	-0.108
			u12							0.447**	-0.013	0.113	-0.102	-0.019	-0.252	-0.056	0.005	-0.143	0.060	-0.128	-0.184	0.085	-0.104	-0.270	-0.006
Game Intelligence		(7) Game Intelligence	All								-0.174	-0.229*	-0.046	-0.008	-0.057	0.060	0.001	0.097	-0.062	-0.147	-0.199	-0.067	-0.193	-0.146	-0.007
			u10							-	-0.015	-0.083	-0.202	-0.055	-0.071	-0.063	-0.081	-0.013	-0.216	0.092	-0.130	0.120	-0.431**	-0.064	-0.001
			u12								-0.417**	-0.414**	0.177	0.041	-0.053	0.175	0.077	0.217	0.140	-0.479***	-0.306*	-0.263	0.160	-0.289	-0.036
Attentional Functioning	ANTI trials	(8) Mean RT ANTI	All									0.428***	-0.517***	0.054	-0.330**	0.020	-0.345**	-0.217*	-0.478***	0.919***	0.337**	0.695***	0.016	0.325**	0.0191
			u10								-	0.229	-0.645***	0.040	-0.568***	0.060	-0.336*	-0.229	-0.628***	0.903***	0.227	0.790***	-0.017	0.028	-0.020
			u12									0.687***	-0.579***	0.127	-0.194	-0.010	-0.370*	-0.352*	-0.630***	0.919***	0.343*	0.742***	-0.056	0.410**	0.178
		(9) Mean RT SD ANTI	All										-0.309**	0.230*	-0.233*	0.070	-0.321**	0.143	-0.396***	0.438***	0.620***	0.480***	-0.116	0.107	0.040
			u10									-	-0.354*	0.234	-0.322*	0.159	-0.339*	0.308*	-0.449**	0.290	0.601***	0.431**	-0.257	-0.182	-0.164
			u12										-0.305*	0.247	-0.165	-0.028	-0.295*	-0.131	-0.447**	0.626***	0.623***	0.577***	0.121	0.361*	0.239
		(10) Mean Error Rate ANTI	All											0.047	0.575***	0.016	0.434***	0.275*	0.866***	-0.634***	-0.197	-0.728***	0.510***	0.089	0.067
			u10										-	0.086	0.661***	0.004	0.471**	0.226	0.903***	-0.770***	-0.336*	-0.748***	0.534***	0.210	0.050
			u12											0.003	0.333*	0.042	0.451**	0.348*	0.774***	-0.651***	-0.056	-0.729***	0.413**	-0.291	-0.076
		(11) Alertness Index RT	All												0.079	0.081	0.075	0.195	-0.029	-0.057	0.159	0.043	0.170	0.119	0.093
			u10											-	-0.006	0.307*	0.006	0.289	0.006	-0.104	0.179	0.022	0.257	0.166	0.082
			u12												0.240	-0.157	0.139	0.093	-0.061	0.036	0.172	0.064	0.042	0.146	0.180
		(12) Alertness Index Error Rate	All													-0.155	0.177	0.236*	0.496***	-0.354***	-0.117	-0.517***	0.188	0.296**	0.180
			u10												-	-0.263	0.348*	0.102	0.628***	-0.575***	-0.319*	-0.648***	0.204	0.310*	0.113
			u12													-0.001	-0.018	0.436**	0.127	-0.198	0.099	-0.311*	0.089	0.144	0.139
		(13) Orienting Index RT	All														-0.098	0.102	-0.012	-0.055	0.131	0.032	0.195	-0.037	-0.003
			u10													-	0.025	0.159	0.048	-0.060	0.292	0.008	0.160	-0.024	0.178
			u12														-0.217	0.041	-0.094	-0.042	-0.044	0.059	0.284	-0.042	-0.244
		(14) Orienting Index Error Rate	All															0.162	0.432***	-0.412***	-0.139	-0.436***	0.356***	-0.184	-0.018
			u10														-	0.170	0.446**	-0.435**	-0.223	-0.378*	0.476**	-0.071	-0.023
			u12															0.171	0.509***	-0.404**	-0.020	-0.509***	0.227	-0.287	0.047
		(15) Control Index RT	All																0.225*	-0.234*	0.109	-0.230*	0.043	-0.000	0.129
			u10															-	0.168	-0.226	0.116	-0.108	0.020	-0.100	0.018
			u12																0.286	-0.382**	0.044	-0.386**	0.055	0.005	0.230
		(16) Control Index Error Rate	All																	-0.581***	-0.271*	-0.756***	0.340**	0.107	0.065
			u10																-	-0.737***	-0.438**	-0.763***	0.429**	0.162	0.004
			u12																	-0.681***	-0.182	-0.796***	0.046	-0.267	-0.095
	VE trials	(17) Mean RT HIT	All																		0.402***	0.722***	-0.180	0.320**	0.189
			u10																	-	0.322*	0.799***	-0.265	-0.009	-0.005
			u12																		0.398**	0.764***	-0.168	0.505***	0.203
		(18) Mean RT SD HIT	All																			0.166	-0.061	0.238*	0.204
			u10																		-	0.212	-0.156	0.047	0.064
			u12																			0.140	0.037	0.281	0.224
		(19) Hits	All																				-0.108	0.016	-0.101
			u10																			-	-0.150	-0.125	-0.130
			u12																				-0.020	0.245	-0.031
		(20) False Alarms	All																					0.071	0.121
			u10																				-	0.129	0.145
			u12																					-0.183	-0.069
	AV trials	(21) AV RT	All																						0.613***
			u10																					-	0.541***
			u12																						0.526***
		(22) AV SD	All																						
			u10																						-
			u12																						

Note: * $p < 0.05$; ** $p < 0.01$; *** $p < 0.001$. Dark grey cells represent BF_{10} higher than 3, indicating moderate to strong evidence for H_1. Medium grey cells represent BF_{10} between 0.33 and 3, indicating lack of evidence for either hypothesis. White cells indicate BF_{10} lower than 0.33, indicating moderate to strong evidence for H_0.
